# Association Between Olfactomedin 4 and Postoperative Prognosis in Patients With Early-Stage Hepatocellular Carcinoma

**DOI:** 10.14309/ctg.0000000000000163

**Published:** 2020-06-18

**Authors:** Wan-Yuan Chen, Yong-Kang Diao, Hang-Dong Jia, Lei Liang, Tian Yang

**Affiliations:** 1 Department of Pathology, Zhejiang Provincial People's Hospital, People's Hospital of Hangzhou Medical College, Hangzhou, Zhejiang, China;; 2 Department of Hepatobiliary, Pancreatic and Minimal Invasive Surgery, Zhejiang Provincial People's Hospital, People's Hospital of Hangzhou Medical College, Hangzhou, Zhejiang, China.

I read with great interest the article by Ye et al., (1) which aimed to examine the prognostic significance of Olfactomedin 4 (OLFM4) in determining recurrence in patients with early-stage hepatocellular carcinoma (HCC). Using the clinical data and tissue specimens (tumor tissue and matched nontumor tissue) of 157 patients who underwent liver resection or liver transplantation, the results of multivariate analysis confirmed OLFM4 cytoplasm staining as an independent predictive factor associated with overall survival in patients with early-stage HCC (hazard ratio: 2.135, 95% confidence interval: 1.135–4.015, *P* = 0.019). Although innovative and inspiring, several points warrant further clarification.

First, the authors mentioned that those patients with early-stage HCC were enrolled in this study. However, they really did not mention the definition of “early stage” in this study. As we know, there have been more than 10 staging systems of HCC worldwide nowadays, including tumor node metastasis staging, the Barcelona Clinic Liver Cancer algorithm, and the Cancer of the Liver Italian Program score, and the Japanese Integrated Staging score (2,3). According to patient's performance status, tumor size and number, vascular invasion, extrahepatic metastasis, and some other related variables, the definitions of early-stage HCC in various staging system are different. Therefore, we wonder to know which staging system did the authors adopt in this study. To our knowledge, if patients with HCC had combined with extrahepatic metastasis, they should be directly divided into advanced-stage HCC in all of these existing HCC staging systems. Surprisingly, as shown in Table 1 of this study, there were 6 (3.8%, 6/151) enrolled patients who had extrahepatic metastasis, who, in our opinion, should be regarded as having advanced-stage HCC but not early-staging HCC. Although only a small number, these 6 patients should be excluded from the analytic cohort in this study as we think.

Second, as for tumor-associated pathological variables of HCC, we appreciate that the authors analyzed tumor grading, presence of vascular invasion, tumor number, and tumor size in their study. However, as we know, apart from these abovementioned variables, they should take other important and commonly used pathological variables into the present study, including the presence of satellite nodules and the presence of complete/incomplete tumor encapsulation, especially for early-stage HCC (4,5). On one hand, the relationship between the expression of OLFM4 and the presence of satellite nodules and tumor encapsulation should be analyzed in this study; on the other hand, these 2 important pathological variables should be added into univariate and multivariate Cox-regression analyses of predictive factors associated with postoperative prognosis in patients with early-stage HCC.

Third, in the Objectives Section of this study, the authors indicated that the purpose of this study was to examine the prognostic significance of OLFM4 in determining recurrence in patients with early-stage HCC. Along this line, the primary endpoint of this study should be recurrence (time-to-recurrence) or recurrence-free survival. As a matter of fact, in this study, the authors used overall survival, but not recurrence-free survival, as the primary endpoint reflecting postoperative prognosis in this study, and they only identified the independent association of OLFM4 with overall survival, but not time-to-recurrence nor recurrence-free survival. Therefore, the conclusions (or the objectives) of this present study should be adjusted or modified according to their current results.

In summary, clarification regarding the abovementioned omissions would greatly solidify the conclusions of the study by Ye et al. (1)

## CONFLICTS OF INTEREST

**Guarantor of the article:** Tian Yang, MD.

**Specific author contributions:** Wan-Yuan Chen, MD, Yong-Kang Diao, MD, and Hang-Dong Jia, MD, contributed equally to this work. W.-Y.C., Y.-K.D., and H.-D.J. participated in the analysis and interpretation of data and in the manuscript drafting. L.L. contributed to the conception. T.Y. contributed to the conception and data interpretation and revised the manuscript for important intellectual content.

**Financial support:** None to report.

**Potential competing interests:** None to report.
